# Analysis of mitochondrial dynamics and function in the retinal pigment epithelium by high-speed high-resolution live imaging

**DOI:** 10.3389/fcell.2022.1044672

**Published:** 2022-10-28

**Authors:** Li Xuan Tan, Jianlong Li, Colin J. Germer, Aparna Lakkaraju

**Affiliations:** ^1^ Department of Ophthalmology, School of Medicine, University of California, San Francisco, CA, United States; ^2^ Department of Cell and Tissue Biology, School of Dentistry, University of California, San Francisco, CA, United States; ^3^ Pharmaceutical Sciences and Pharmacogenomics Graduate Program, University of California, San Francisco, CA, United States; ^4^ Department of Anatomy, School of Medicine, University of California, San Francisco, CA, United States

**Keywords:** mitochondria, live imaging, retina, RPE, pigmented and albino mice

## Abstract

Mitochondrial dysfunction is strongly implicated in neurodegenerative diseases including age-related macular degeneration (AMD), which causes irreversible blindness in over 50 million older adults worldwide. A key site of insult in AMD is the retinal pigment epithelium (RPE), a monolayer of postmitotic polarized cells that performs essential functions for photoreceptor health and vision. Recent studies from our group and others have identified several features of mitochondrial dysfunction in AMD including mitochondrial fragmentation and bioenergetic defects. While these studies provide valuable insight at fixed points in time, high-resolution, high-speed live imaging is essential for following mitochondrial injury in real time and identifying disease mechanisms. Here, we demonstrate the advantages of live imaging to investigate RPE mitochondrial dynamics in cell-based and mouse models. We show that mitochondria in the RPE form extensive networks that are destroyed by fixation and discuss important live imaging considerations that can interfere with accurate evaluation of mitochondrial integrity such as RPE differentiation status and acquisition parameters. Our data demonstrate that RPE mitochondria show localized heterogeneities in membrane potential and ATP production that could reflect focal changes in metabolism and oxidative stress. Contacts between the mitochondria and organelles such as the ER and lysosomes mediate calcium flux and mitochondrial fission. Live imaging of mouse RPE flatmounts revealed a striking loss of mitochondrial integrity in albino mouse RPE compared to pigmented mice that could have significant functional consequences for cellular metabolism. Our studies lay a framework to guide experimental design and selection of model systems for evaluating mitochondrial health and function in the RPE.

## Introduction

Mitochondria are essential signaling platforms that regulate ATP generation, calcium homeostasis, and cell fate decisions. This functional versatility is made possible by the continuous remodeling of the mitochondrial network by fusion, fission, and mitophagy, and communication with other organelles *via* membrane contacts that help the cell adapt to diverse metabolic needs (Gordaliza-Alaguero et al., 2019; Giacomello et al., 2020). Mitochondrial fission generates reactive oxygen species (ROS) and activates mitophagy whereas mitochondrial fusion into tubular networks prevents mitochondrial DNA (mtDNA) damage, improves the efficiency of oxidative phosphorylation (OXPHOS), and enhances interactions with the endoplasmic reticulum (ER) necessary for Ca^2+^ flux (Giacomello et al., 2020).

In the eye, mitochondrial dysfunction is strongly associated with several diseases including inherited and age-related macular degenerations (AMD), which cause irreversible vision loss in millions of people globally (Handa et al., 2019; Ferrington et al., 2021). A key site of insult in these diseases is the postmitotic retinal pigment epithelium (RPE), which sits between the photoreceptors and the choriocapillaris, and performs numerous functions essential for maintaining healthy vision (Caceres and Rodriguez-Boulan, 2020; Lakkaraju et al., 2020). Clinically, RPE abnormalities and the accumulation of lipid-protein aggregates called drusen above and beneath the RPE are associated with AMD progression.

The RPE is a highly metabolically active tissue that relies on mitochondrial OXPHOS for its energy needs. It shuttles glucose to the photoreceptors and muller glia, which rely on aerobic glycolysis, and uses metabolites from glycolysis and photoreceptor outer segment clearance as OXPHOS substrates (Hurley, 2021). In mice, disruption of this “metabolic ecosystem” (Du et al., 2016; Kanow et al., 2017; Reyes-Reveles et al., 2017) by RPE-specific deletion of mitochondrial genes causes a glycolytic shift in the RPE, which starves photoreceptors and eventually leads to retinal degeneration (Zhao et al., 2011; Brown et al., 2019). These studies highlight the central role of RPE mitochondria in regulating the health of the retina and suggest that RPE mitochondrial injury can trigger photoreceptor dysfunction and vision loss.

Increased mtDNA damage and OXPHOS defects (Ferrington et al., 2017; Zhang et al., 2020) have been documented in RPE cultures established from human donors with AMD. Using high-resolution imaging, we recently demonstrated that RPE mitochondria in retinal cryosections from AMD donors are highly fragmented, in contrast to the interconnected, healthy mitochondria seen in the RPE of unaffected donors (La Cunza et al., 2021). High-speed live imaging of cell-based and mouse model of macular degeneration helped us identify a novel biophysical mechanism that directly connects RPE mitochondrial dysfunction to drusen formation: redox state changes in fragmented mitochondria drive liquid-liquid phase separation of proteins with reactive cysteines, which nucleate drusen-like aggregates within the RPE (La Cunza et al., 2021). Thus, RPE mitochondrial deficits not only compromise RPE homeostasis but also initiate the development of AMD-associated pathologies.

These studies underscore the importance of analyzing mitochondrial form and function to better understand retinal biology and pathological mechanisms and to identify novel drug targets for diseases like AMD. Here, we demonstrate the power of high-speed, high-resolution live-cell imaging to evaluate mitochondrial dynamics in the RPE. We discuss important considerations when selecting imaging modalities and issues specific to the RPE that can interfere with mitochondrial imaging such as fixation, pigmentation, and lipofuscin accumulation. Lastly, by extending this technology to image mitochondrial dynamics in the living mouse RPE, we demonstrate that mitochondrial morphology, connectivity, and subcellular distribution are strikingly altered in albino mice RPE compared to that in pigmented mice, which could have significant functional consequences on cellular metabolism. Our studies lay a framework to guide experimental design and selection of model systems for evaluating mitochondrial health and function in the RPE.

## Materials and methods

### Primary RPE cultures and transfections

Primary RPE were harvested from porcine eyes as previously described (Toops et al., 2014), plated onto T25 flasks in DMEM with 10% FBS for the first week and 1% FBS for the second week (American Type Culture Collection, Manassas, VA). To generate polarized cultures, cells were plated at ∼350,000 cells/cm^2^ onto collagen-coated Transwell filters (Corning, Corning, NY) (Toops et al., 2014). Primary RPE were magnetofected with 0.5 µg mito-GCaMP5G plasmid (105,009, Addgene, Watertown, MA) for every ∼3 × 10^5^ cells using LipoMag Transfection Kit (OZ Biosciences, San Diego, CA).

### Preparation of mouse RPE flatmounts for live imaging

All animal experiments were approved by the Institutional Animal Care and Use committee at the University of California, San Francisco. 3- to 5-months-old pigmented 129S1/SvlmJ (Jackson Laboratories), C57BL/6J (Jackson Laboratories), and albino BALB/c (Knox lab, UCSF) mice were raised under 12-h cyclic light with standard lab diet. We used 3 mice per strain, 1 eye of each mouse, therefore n = 3 biological replicates for each experiment. Mouse eyes were dissected immediately after CO_2_ asphyxiation and enucleation. Extra-ocular tissues were removed under a dissection microscope, and a hole was made near the ora serrata with a 25G x 1 ½ BD PrecisionGlide (BD Biosciences, 305,127). A pair of curved spring micro-scissors and a tweezer was used to remove the anterior portion and the lens from the eye, leaving behind the eyecup. Four deep, perpendicular relaxing cuts were made. The retina was then peeled and removed by snipping it off at the optic nerve head. The perpendicular cuts were extended to obtain four RPE flatmount leaflets, which were then incubated in complete growth media (DMEM supplemented with 1% FBS, 1% NEAA, 1% penicillin/streptomycin) at 37°C in a cell culture incubator. Each leaflet was stained and imaged as described below. Dissection was performed in dim light for albino mice.

### High-speed high-resolution live imaging system

Mouse RPE flatmounts or primary RPE cultures were stained with Mitotracker (200 nM, 15 min), ERtracker (1 µM, 30 min), Biotracker ATPRed (10 µM, 15 min), or tetramethyl rhodamine (TMRE) (500 nM, 10 min) in recording media (1 x HBSS, 4.5 g/L glucose, 0.01 M HEPES), rinsed, and mounted onto a Warner chamber as previously described (Toops et al., 2014). Briefly, RPE grown on Transwell filters were excised using a scalpel blade and mounted cell-side down onto ∼35 µL of recording medium between two coverslips. For RPE grown on glass-bottom dishes (Mattek), growth media was replaced with recording media immediately before imaging. Images were acquired at 37°C in Okolab humidified environmental chamber using CFI60 Apochromat TIRF ×100 oil immersion objective (1.49 NA). Live imaging was performed on the Nikon spinning disk confocal microscope equipped with: Yokogawa CSU-X1 confocal spinning disk head, Nikon Eclipse Ti2-E inverted microscope, Live-SR super-resolution module, Andor iXon Ultra 888 EMCCD camera, TI2-S-SE-E motorized stage with piezo-Z for rapid Z-stack acquisition, and a laser combiner with four solid-state lasers at 405, 488, 561, and 640 nm and the corresponding band-pass emission filter sets (Chroma) loaded on a FLI high speed filter wheel. The Live-SR is a super-resolution module that increases x-y resolution to ∼ 120–140 nm, making it comparable to the resolution achieved by structured illumination microscopy (SIM). During image acquisition, care was taken to maintain the same laser power, exposure and electron-multiplying gain settings. Movies were acquired at the following rates: Supplementary Movies S1 (∼2 s intervals for 2 min), Supplementary Movies S2 (∼15 s intervals for 2 min), Supplementary Movies S3 (5 s intervals for 1 min), Supplementary Movies S4 (no delay (∼3.7 s intervals for a z-stack) for 5 min), Supplementary Movies S5 (15 s intervals for 5 min), Supplementary Movies S9 (10 s intervals for 3 min).

### Image analysis

Images were subjected to Gaussian filtering and background subtraction prior to analysis using the same thresholds for all images. Imaris 9.9 (Bitplane) was used for surface rendering using the “Surfaces” module to determine parameters such as mean intensity, object volumes, and number of discrete objects. For quantification of mitochondrial volumes, thresholds for surface creation were guided by automatic thresholding. For timelapse analyses, tracking was enabled to track intensity changes with time, and intensity values were exported from the statistics tab. For ATP analysis, surfaces were created for the ATP channel as detailed above. The number of ATP puncta was exported from the statistics tab and normalized to Mitotracker intensity in Microsoft Excel. TMRE signals were pseudo-colored with the “Fire” look-up-table under the “Mapped Color” tab to visualize relative intensity. NIS Elements (Nikon) was used for automatic deconvolution of images, generation of kymographs, and line intensity profiles. Insets for kymographs and line intensity profiles were exported from NIS Elements using the “Create View Snapshot” function. All exported data were compiled on Microsoft Excel and plotted on Prism 8 (GraphPad).

### Immunostaining and imaging

Primary porcine RPE monolayers were fixed with 2% PFA for 10 min at room temperature. After brief rinses in 1x PBS, cells were incubated with blocking solution (1% BSA in PBS) for 30 min at room temperature, followed by 1 h incubation with CoraLite 488-conjugated TOM20 primary antibody (see Supplementary Table S1) diluted in 1% BSA in the presence of 0.1% saponin for permeabilization. Cells were then rinsed and mounted in Vectashield. For immunostaining mouse RPE flatmounts, retinas were removed and the flatmounts were fixed with 4% PFA for 1 h at room temperature, rinsed, and blocked for 1 h in 2% BSA in PBS in the presence of 0.1% Triton-X for permeabilization. Flatmounts were then stained with TOM20 primary antibody, followed by AlexaFluor secondary antibody, phalloidin, and DAPI (see Supplementary Table S1), sealed under no. 1.5 coverslips using Vectashield as a mounting medium. All images were captured on Nikon spinning disk confocal microscope using 100×/1.49 NA oil objective. Images were subjected to background subtraction and Gaussian smoothing in Imaris (Bitplane, South Windsor, CT).

### Statistical analysis

Data were analyzed by either unpaired two-tailed *t*-test with Welch’s correction for testing effects between two experimental groups or one-way ANOVA with post-hoc analysis for comparison between multiple groups (GraphPad Prism). Specific tests, sample sizes, and p-values are included in the legend of each figure.

## Results

### Dynamic RPE mitochondrial networks are best captured by high-speed live imaging and are destroyed by fixation

In all cells and tissues including the RPE, mitochondria undergo coordinated fusion and fission to establish extensive networks that span the volume of the cell. The integrity of the mitochondrial network is directly related to mitochondrial health and function. For instance, mutations in proteins that regulate fusion and fission cause diseases characterized by fragmentation of the mitochondrial network, loss of mitochondrial membrane potential, and OXPHOS defects (Giacomello et al., 2020). In the context of AMD, abnormal complement activation at the RPE cell surface leads to increased intracellular calcium that activates mitochondrial fission machinery resulting in mitochondrial fragmentation and production of reactive oxygen species in the RPE (Tan et al., 2016). Therefore, changes in mitochondrial morphology reflect changes in mitochondrial function.

**FIGURE 1:**
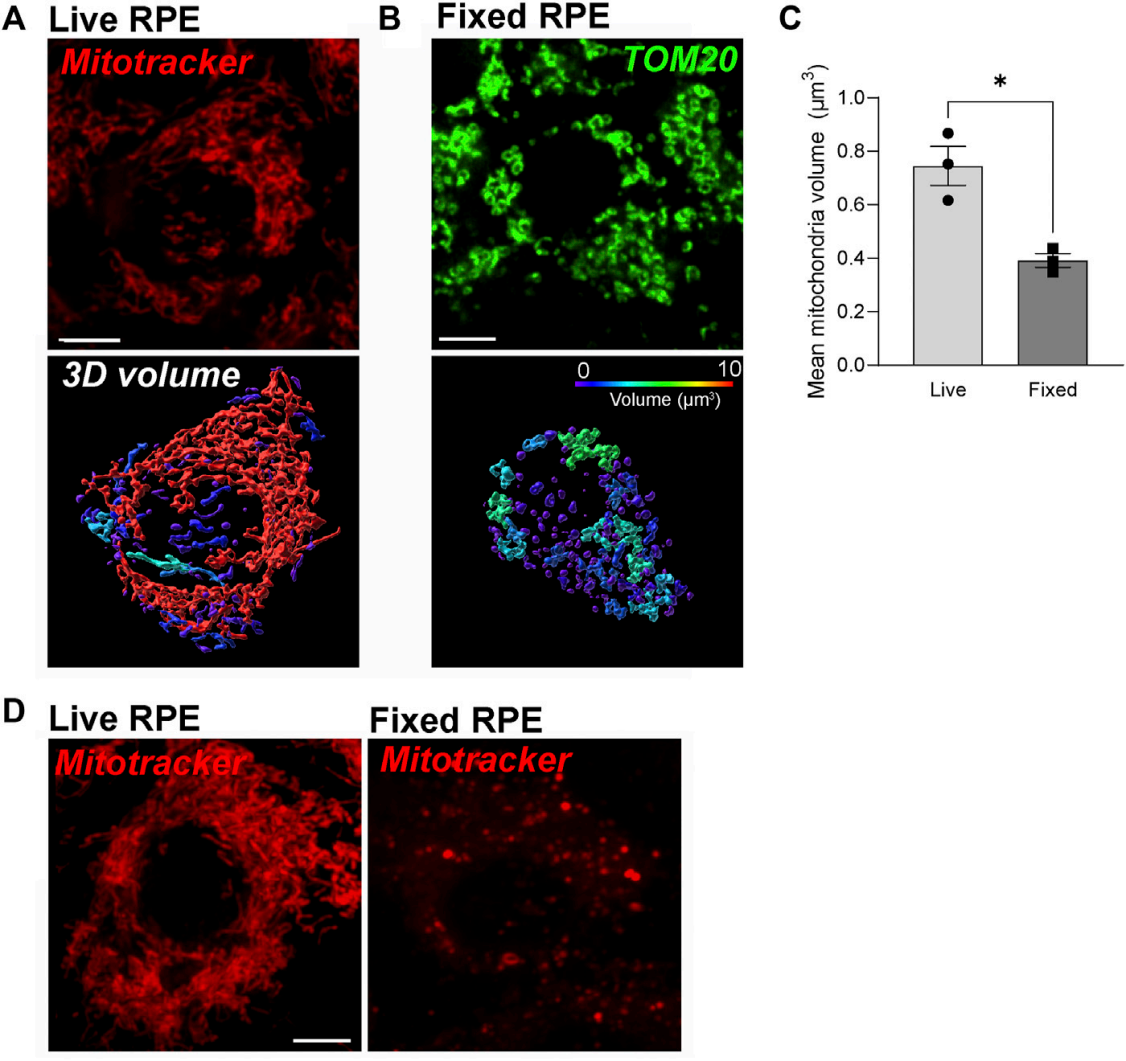
Dynamic RPE mitochondrial networks are best captured by high-speed live imaging and are destroyed by fixation. **(A)** Stills from high-speed high-resolution live imaging of primary RPE monolayers labeled with Mitotracker deep red (red). **(B)** Immunofluorescence imaging of paraformaldehyde-fixed primary RPE monolayers stained with an antibody to TOM20 (green). In A and B, top panel: single-plane view; Lower panel: 3D reconstructions. Warmer colors indicate larger volumes or integrated mitochondria, cooler colors indicate smaller volumes or fragmented mitochondria. **(C)** Quantification of mitochondrial volumes in A and **(B)**. Mean ± SEM. *n* = 3 independent experiments, ∼170 cells per condition. *p = 0.0286 with Welch’s t-test. **(D)** Comparison of Mitotracker deep red (red) fluorescence before and after fixation.

Immunostaining for mitochondrial proteins is widely used to examine mitochondrial morphology in fixed tissues or cells. Chemical fixation using paraformaldehyde or other fixatives is known to alter the morphology of endosomes and lysosomes and destroy tubular endosomes (Murk et al., 2003). To investigate how fixation affects RPE mitochondria, we compared 3D reconstructions of mitochondrial networks in the RPE generated from live imaging with those from immunofluorescence images of fixed RPE monolayers. Mitochondria in polarized primary RPE monolayers were labeled with Mitotracker Deep Red (Thermo Fisher) and imaged live using the Nikon spinning disk confocal microscope with a Live-SR module (See Materials and Methods for a detailed description of the imaging system and image analysis). Cells were then fixed and stained with an antibody to TOM20 and imaged using the same camera and objective as for live imaging. Our live-cell imaging showed that the mitochondrial network in the RPE is filamentous and highly interconnected as has been reported in live imaging studies of other cell types (Rambold et al., 2011) (Figure 1A). However, we observed that even light fixation in 2% paraformaldehyde for 10 min almost completely dismantles this network, indicating that RPE mitochondria are highly susceptible to fixation-induced artifacts (Figures 1B,C). These data strongly suggest that immunostaining-based analysis of mitochondrial integrity in the RPE should be corroborated with live imaging studies whenever possible.

In our experience, dyes that emit at longer wavelengths such as Mitotracker Deep Red (Excitation 644 nm; Emission 665 nm) are best for imaging pigmented tissues like the RPE. This is because melanin quenches the fluorescence of shorter wavelength dyes, especially those that emit in the 420–520 nm range. Although Mitotracker Deep Red FM (Thermo Fisher) has been reported to be retained after fixation, we noticed a significant loss of fluorescence intensity after fixation (Figure 1D).

### Mitochondrial integrity is coupled to RPE polarity and differentiation status

**FIGURE 2:**
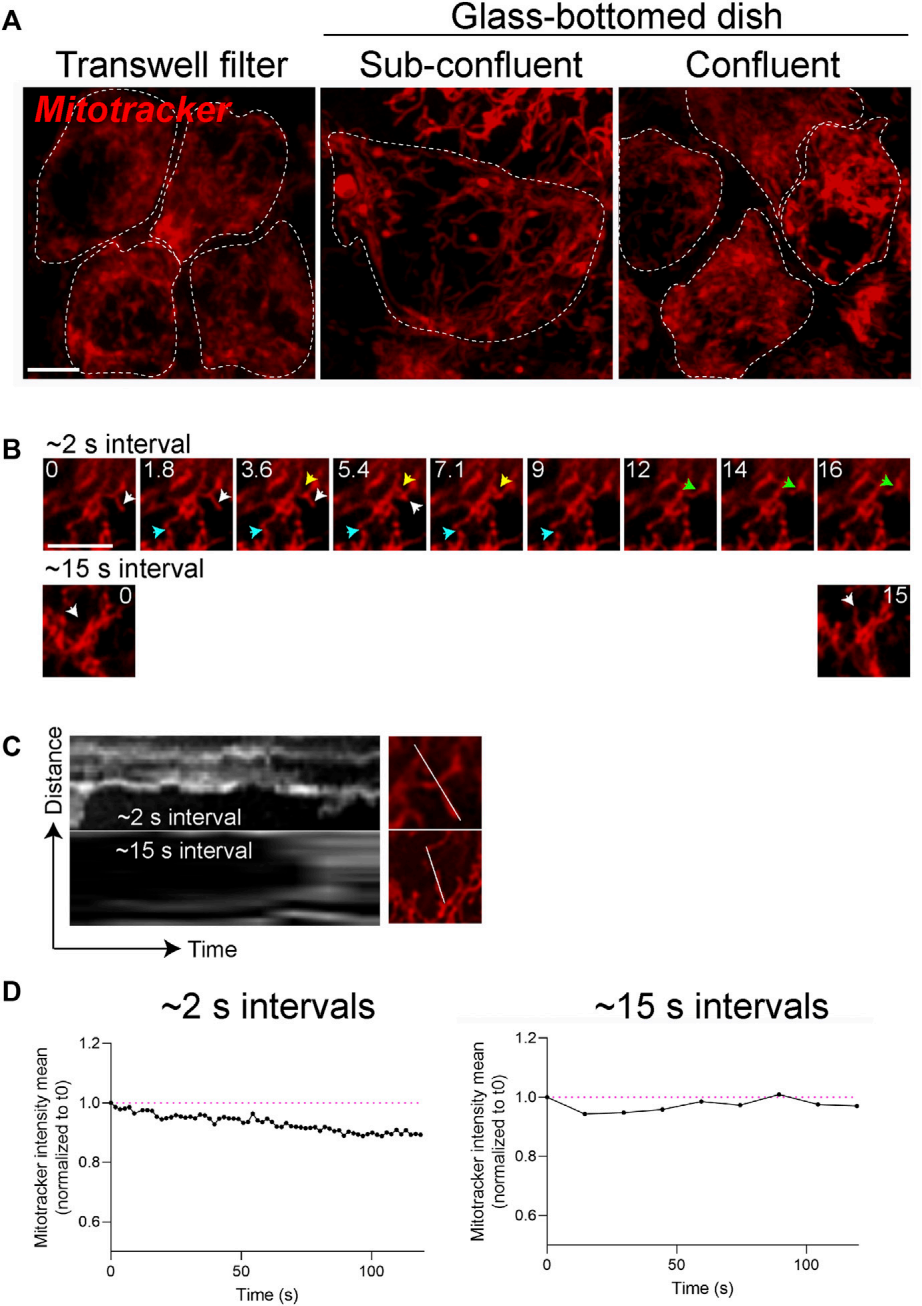
Mitochondrial integrity is coupled to RPE polarity and differentiation status. **(A)** Stills from live imaging of mitochondrial networks (Mitotracker, red) in primary RPE grown on Transwell filters to enable differentiation and establishment of polarity or on glass-bottomed dishes. Cell boundaries demarcated by white dotted lines show packing density or confluence of the monolayer. Scale bar = 5 µM. **(B)** High magnification stills from live imaging of mitochondria acquired with ∼2 s intervals (top panel) or at 15 s intervals (bottom panel). Only the first ∼16 s are shown. Blue, yellow, and white arrows depict distinct fusion or fission events. Scale bar = 5 µM. **(C)** Kymographs of mitochondrial movements along the indicated axis. Also see Supplementary Movies S1, S2. **(D)** Photobleaching and loss of signal intensity as a function of acquisition speeds. Magenta lines mark signal intensities at the start of imaging (t = 0).

The terminally differentiated polarized phenotype of the RPE–with a distinct repertoire of proteins on the apical and basolateral membranes (Caceres and Rodriguez-Boulan, 2020)—is central to its functional specializations. Recent studies suggest that mitochondrial metabolism helps maintain the differentiated status of the RPE (Hazim et al., 2022). To investigate if this is a bidirectional relationship - i.e., whether RPE polarity also modulates mitochondrial integrity - we compared mitochondrial networks in polarized adult primary RPE monolayers cultured on collagen-coated semipermeable polyester Transwell filters (Toops et al., 2014) with those in adult primary RPE cultured on glass coverslips. Live imaging showed that polarized RPE on filters have a more complex mitochondrial reticulum relative to those on glass. However, increasing the plating density of RPE on glass increases mitochondrial connectivity to a level comparable to RPE on filters (Figure 2A). This is because plating RPE at high density helps establish a confluent monolayer by preventing cell division and thereby promoting differentiation (Lane et al., 2014; Toops et al., 2014). These data support the hypothesis that organization of the mitochondrial network is tightly coupled to the differentiation status of the RPE. Collagen or other extracellular matrix components used to culture primary RPE could also contribute to mitochondrial integrity *via* integrins or by modulating the actin or microtubule cytoskeleton to regulate mitochondrial fission and fusion (Rizzuto, 2003).

One technical challenge in working with polarized RPE monolayers is their very low transfection efficiency and expression of exogenous genes. In addition to dyes such as Mitotracker, viral transduction using baculovirus to express fluorescently-tagged pyruvate dehydrogenase (CellLight Mitochondria, Thermo Fisher) can be used for live imaging (Table 1). Transducing primary polarized RPE with CellLight Mitochondria at ∼14 virus particles/cell for 18 h yielded transduction efficiencies of ≥ 40% with no observed toxicity and labeled mitochondria comparable to that seen with Mitotracker probes (Tan et al., 2016; La Cunza et al., 2021). Compared to transgene expression, fluorescent probes that label the mitochondria uniformly label almost all the cells in the monolayer. However, genetic approaches yield longer-lasting labeling and are relatively non-toxic compared to fluorescent dyes. Studies in breast cancer cell lines found that Mitotracker deep red inhibits basal respiration and blocks ATP synthesis at concentrations at or above 500 nM (Sargiacomo et al., 2021). Although the toxicity of mitochondria dyes is likely cell-type dependent, care must be taken to avoid artifacts by using the lowest-possible concentration for labeling.

**TABLE 1:** Tools to visualize mitochondria in polarized RPE monolayers.

	Fluorescent probes	Transgenes
Ease of use	Very easy, ∼15 min labeling time	Easy, overnight transfection/transduction
Efficiency of labeling	Very high, almost all cells labelled	∼30–50% depending on the constructs used
Stability of signal	High	High
Imaging window	Must be imaged immediately after labeling	Days
Toxicity	Reported to be toxic at high concentrations	Minimal observed or reported

Because mitochondria are highly dynamic, capturing rapid fission and fusion events necessitates the use of microscopes with high spatial and temporal resolution. The acquisition speed of timelapse experiments needs to be optimized for imaging the trafficking and fission/fusion dynamics of mitochondria. Compared to the fission and fusion events captured with timelapse imaging acquired with ∼2-second intervals, acquisitions at 15-second intervals captured significantly fewer movements (Figures 2B,C, Supplementary Movies S1, S2). Choosing the acquisition speed needs to be balanced with other experimental requirements such as imaging entire volume of the cell (z-stacks), single- or multi-channel imaging, and the susceptibility of the fluorescence probe to photobleaching. For instance, although not significant, acquiring images at 2-second intervals results in a slight loss of Mitotracker signal intensity after 2 min compared to when images are acquired at 15-second intervals (Figure 2D). Thus, RPE polarity, choice of reporter, and acquisition speed are important parameters that can impact live imaging of mitochondrial dynamics.

### Live imaging reveals morphological and functional heterogeneity of mitochondria in the RPE

**FIGURE 3:**
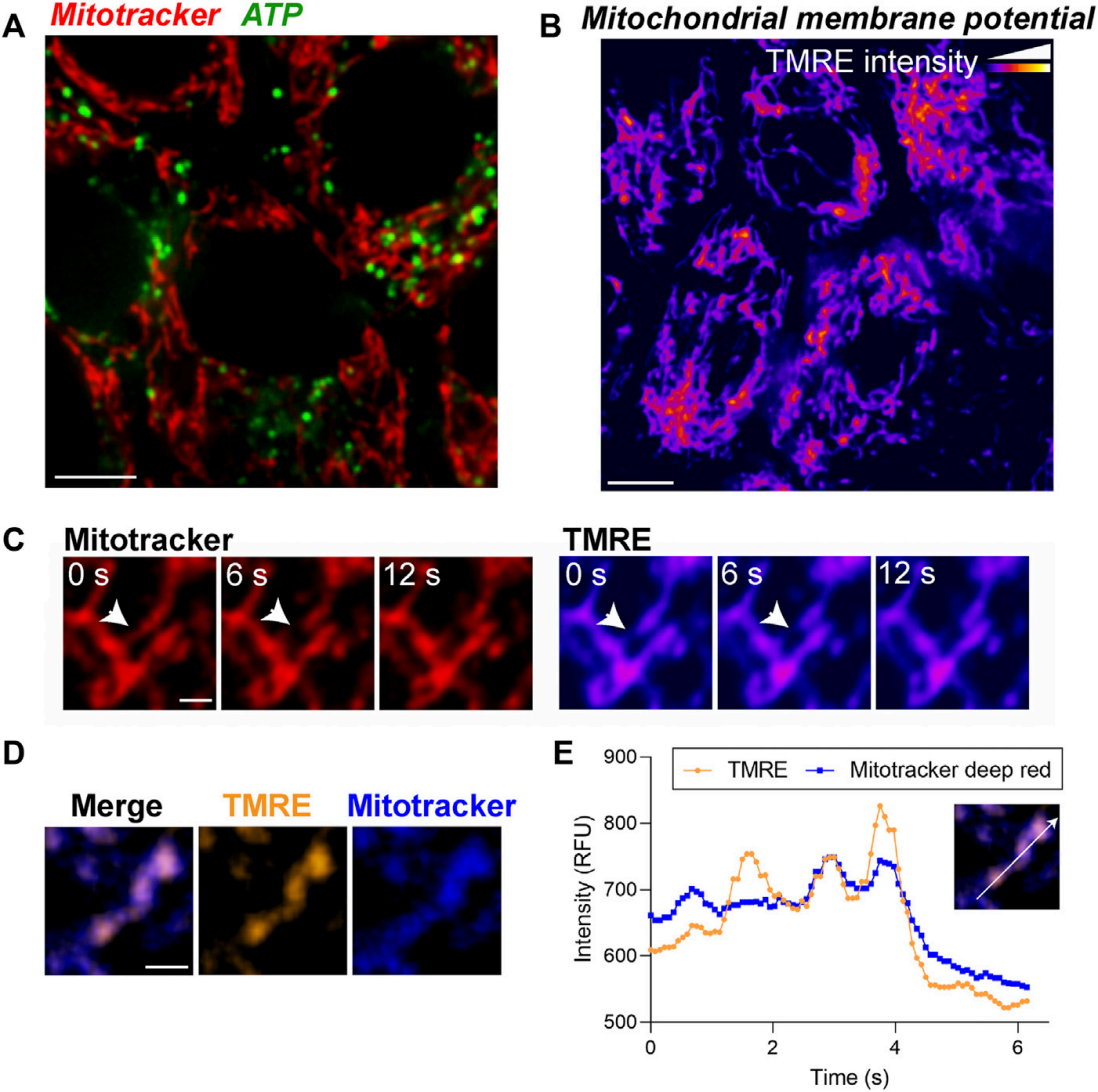
Live imaging of ATP and mitochondrial membrane potential demonstrates morphological and functional heterogeneity of mitochondria in the RPE. **(A)** Stills from live imaging of mitochondria (Mitotracker, red) and BioTracker-ATP (green) in primary RPE monolayers. **(B)** Mitochondrial membrane potential (MMP) measured by TMRE labeling in primary RPE monolayers. Color scale: warmer colors indicate higher TMRE intensities. **(C)** High magnification images showing local heterogeneity of MMP within a single mitochondrion. Note that areas of low TMRE intensity predict putative fission sites (arrow). Scale bar = 1 µM. Also see Supplementary Movies S3. **(D)** Overlay of TMRE (gold) and Mitotracker deep red (blue). Note that TMRE is more sensitive to local MMP heterogeneity within a single mitochondrion compared to Mitotracker deep red. Right panels show split channel views. Scale bar = 1 µM. **(E)** Intensity profile of TMRE and Mitotracker deep red along the axis indicated in the inset.

Although Mitotracker and CellLight constructs are useful for examining the integrity and dynamics of the mitochondrial network, analysis of specific mitochondrial functions requires specialized tools to quantify ATP release, mitochondrial membrane potential, and calcium flux (Iannetti et al., 2019). Altered expression of the mitochondrial ATP synthase has been reported in neurodegenerative diseases including AMD (Nordgaard et al., 2008; Ebanks and Chakrabarti, 2022) but whether and to what extent these changes translate to impaired mitochondrial ATP production has yet to be established. To evaluate ATP generation in the RPE in realtime, we used BioTracker ATP-Red (Millipore). The probe forms a non-fluorescent closed-ring structure in the absence of ATP; however, the covalent bonds holding the ring are broken by ATP, resulting in fluorescence unquenching (Wang et al., 2016). Primary RPE monolayers labeled with Mitotracker and BioTracker ATP showed ATP puncta along mitochondrial filaments (Figure 3A). Intra- and intercellular heterogeneity in ATP levels can be quantified by measuring the number and intensity of puncta and normalizing to either cell number or Mitotracker intensity (Rieger et al., 2021).

As mitochondrial ATP production is dependent on the electrochemical gradient generated by the mitochondrial membrane potential (ΔΨm), changes in ΔΨm are an important indicator of mitochondrial health and function. Tetramethylrhodamine ethyl ester (TMRE) is widely used to measure ΔΨm as changes in TMRE fluorescence positively correlate with ΔΨm. In primary RPE monolayers, on a cellular level, the TMRE fluorescence pattern was comparable to that of Mitotracker (Figure 3B). However, at a subcellular level, we observed local changes in TMRE fluorescence within a single mitochondrion that preceded mitochondrial scission (Figure 3C, Supplementary Movies S3), suggesting that changes in ΔΨm mark putative scission sites (Twig et al., 2008). Although the mitochondrial localization of Mitotracker Deep Red is also dependent on ΔΨm, our live imaging data show that in primary RPE monolayers, TMRE staining is more sensitive than Mitotracker Deep Red in capturing subtle changes in ΔΨm (Figures 3D,E).

### Functional relevance of mitochondria-ER and mitochondria-lysosome contacts in the RPE

Mitochondria form elaborate contacts with other organelles including the ER and lysosomes that act as conduits for exchanging calcium, lipids, and metabolites (Rieusset, 2018; Aoyama-Ishiwatari and Hirabayashi, 2021; Cisneros et al., 2022). The architecture and function of these contacts has been largely studied in cell lines, which has little relevance to the postmitotic RPE. As these contacts are continuously remodeled in response to the changing metabolic needs of the local environment within the cell, high-speed live imaging is essential to capture contact sites and follow their functions. Here, we took advantage of the availability of probes that selectively label the ER (ER-tracker), mitochondria (Mitotracker), and lysosomes (LysoTracker) and the high speed of acquisition of the spinning disc imaging system to capture ER-mitochondrial calcium transport and lysosome-mediated mitochondrial fission in polarized primary RPE monolayers.

**FIGURE 4:**
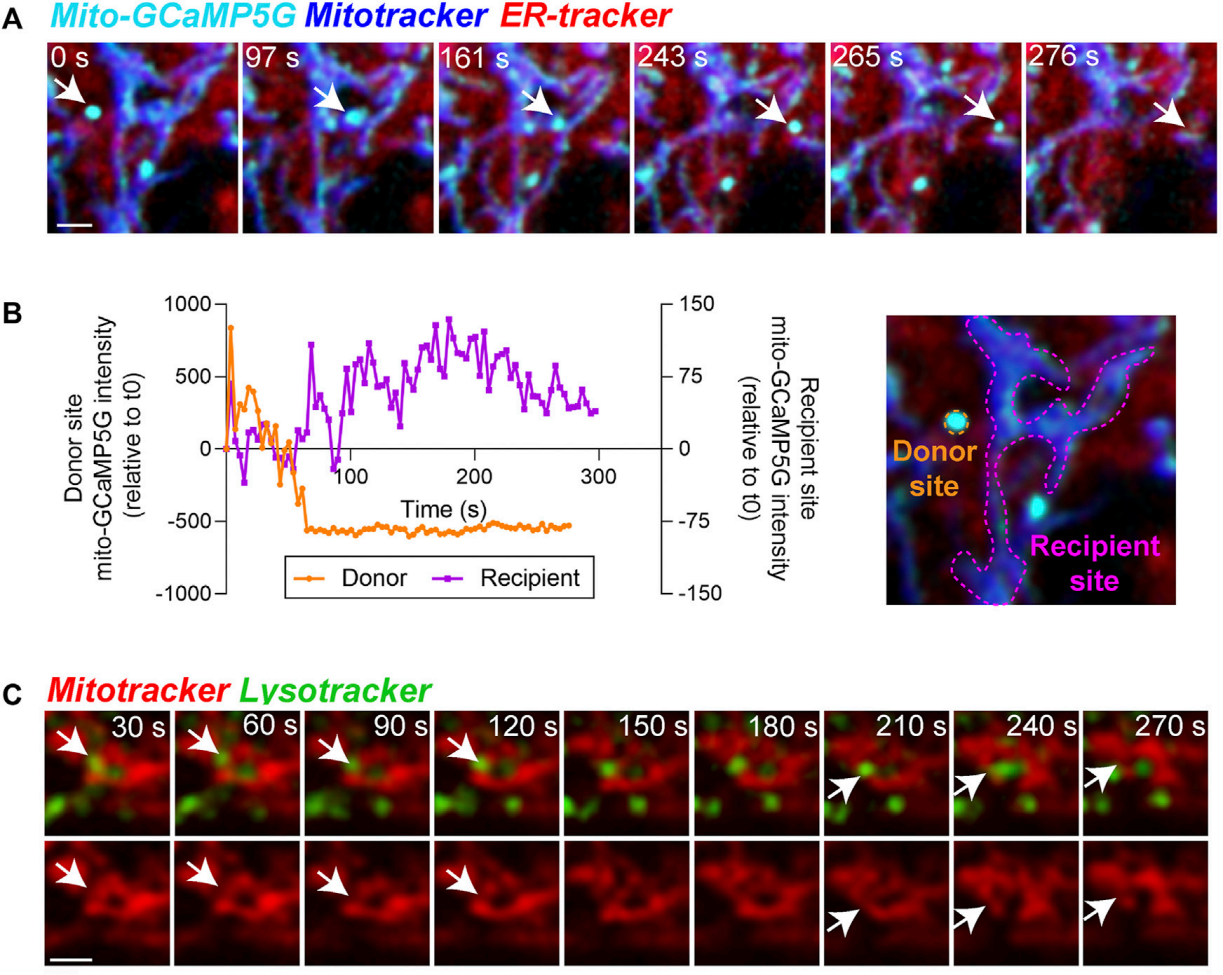
Functional relevance of mitochondria-ER and mitochondria-lysosome contacts in the RPE. **(A)** Stills from live imaging of interactions between mitochondrial calcium (mito-GCaMP5G, cyan), ER-tracker (red), and Mitotracker deep red (blue) in primary RPE monolayers showing heterogeneity of mitochondrial calcium within the cell. Arrow indicates one calcium transfer event. Note ER-mediated transfer of calcium to mitochondria in frames 243 s and 265 s. Images were deconvolved using Nikon NIS-Elements. Scale bar = 1 µM. Also see Supplementary Movies S4. **(B)** Changes in mean intensity of mito-GCaMP5G on the indicated donor and recipient sites. **(C)** Stills from live imaging of contacts between Mitotracker (red) and Lysotracker (green). Top panel shows merged view, bottom panel shows Mitotracker signal only. Arrows indicate mitochondrial fission events. Images were deconvolved using Nikon NIS-Elements. Scale bar = 1 µM. Also see Supplementary Movies S5.

Calcium transport from the ER to mitochondria regulates critical mitochondrial functions. The production of ATP in the mitochondria, for instance, is driven by calcium-sensitive mitochondrial enzymes (Griffiths and Rutter, 2009). However, mitochondrial calcium overload induces fragmentation by activating fission machinery including dynamin-related protein 1 (DRP1) (Horn et al., 2020) and can also lead to cell death by activating the mitochondrial permeability transition pore (Bauer and Murphy, 2020). To evaluate focal changes in mitochondrial calcium, we transfected the RPE with the mitochondria-targeted fluorescent calcium sensor (mito-GCaMP5G) (Kwon et al., 2016). Our live imaging data revealed a heterogeneous distribution of calcium within the mitochondrial network (Figure 4A) and mitochondria-ER contact sites mediating dynamic transport of calcium between mitochondria (Figure 4B, Supplementary Movies S4).

Apart from extensive contacts with the ER, mitochondria also interact dynamically with lysosomes. These mitochondria-lysosome interactions serve many important functions, including mediating substrate transfer and maintaining mitochondrial homeostasis through autophagic clearance (mitophagy) (Giacomello et al., 2020; Cisneros et al., 2022). Recruitment of mitochondrial fission machinery to mitochondria-lysosome contact sites has been shown to promote mitochondrial fission in several cell lines (Wong et al., 2018; Qiu et al., 2022). High resolution imaging of interactions between mitochondria and lysosomes in primary RPE monolayers revealed multiple dynamic interactions between the two organelles that precede mitochondrial fission events (Figure 4C, Supplementary Movies S5).

### Live imaging of mouse RPE flatmounts reveals an unexpected impact of genetic background on mitochondrial integrity and function

Investigating mitochondrial dynamics in the intact living mouse retina is essential for understanding how RPE mitochondrial function and dysfunction contributes to retinal disease. Mice, which are widely used to model and study retinal degenerations, are generated on different genetic backgrounds such as the pigmented 129S1/SvlmJ and C57BL/6J mice, the BALB/c albino mice, as well as mixed backgrounds (Zhao et al., 2011; Mao et al., 2014; Biswal et al., 2018; Brown et al., 2019; La Cunza et al., 2021). The RPE in its native state is pigmented due to the presence of melanosomes, which protect the RPE by scavenging free radicals (Rózanowski et al., 2008). Consequently, transgenic mice on the albino BALB/c background have been used to investigate mitochondrial dysfunction in the RPE as they are more susceptible to photo-oxidative stress (Biswal et al., 2018; Brown et al., 2019). However, to compare studies on mitochondrial proteins in the RPE of transgenic mice of different backgrounds, it is important to have comparative data on mitochondrial morphology and function in the RPE of wildtype pigmented and albino mouse strains.

**FIGURE 5:**
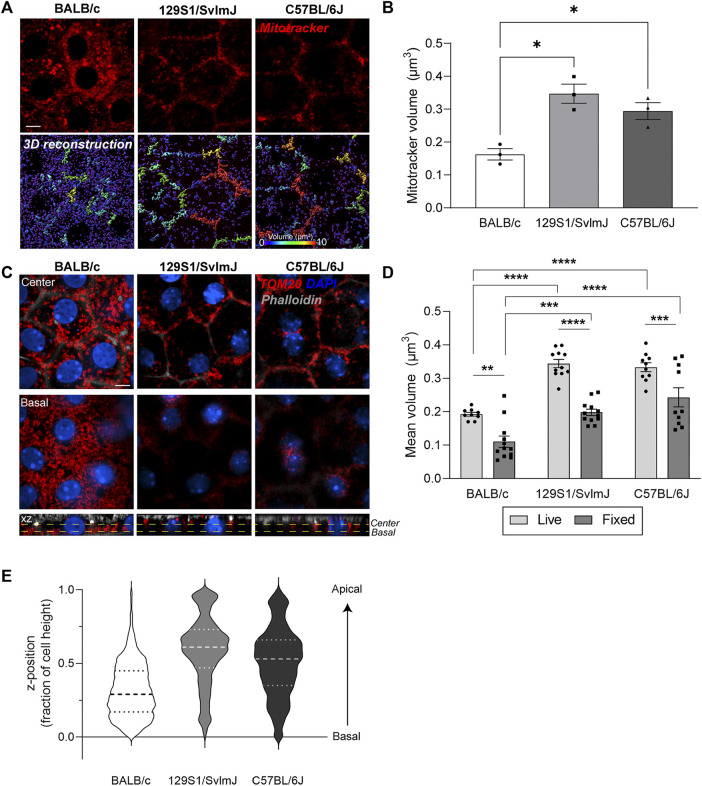
Live imaging of mouse RPE flatmounts reveals impaired mitochondrial integrity in albino mouse RPE. **(A)** Stills from live imaging of Mitotracker deep red (red) in RPE flatmounts from albino BALB/c, pigmented 129S1/SvlmJ, and pigmented C57BL/6J mice. Lower panel: 3D reconstructions of mitochondria. Scale bar = 5 µM. Warmer colors indicate larger volumes or integrated mitochondria, cooler colors indicate smaller volumes or fragmented mitochondria. **(B)** Quantification of mitochondrial volumes in **(A)**. Mean ± SEM. *n* = 3 mice per strain. *p = 0.0215 between BALB/c and 129S1/SvlmJ; *p* = 0.0425 between BALB/c and C57BL/6J by one-way ANOVA. **(C)** Immunofluorescence images of paraformaldehyde-fixed mouse RPE flatmounts stained for TOM20 (red). Top: Single-plane view across the center of the RPE; Middle: Single-plane view across the basal side of the RPE; Lower panel: x-z view showing mitochondrial distribution along the height of the RPE. Phalloidin (grey) labels the actin cytoskeleton and microvilli at the apical surface of the RPE. Also see Supplementary Movies S6–8. **(D)** Quantification of mitochondrial volumes in RPE flatmounts from live and fixed imaging. Mean ± SEM., **, *p* = 0.023; ***, *p* = 0.0009; ****p < 0.0001. Two-way ANOVA was used for comparison between live and fixed imaging, one-way ANOVA was used for comparison within live and fixed imaging respectively. Representative data from ∼60 cells per mouse, data based on *n* = 3 mice per strain. **(E)** Representative violin plot of individual mitochondria z-positions normalized to cell height (0 = bottom-most z-position, 1 = top-most z-position). Dashed lines represent quartiles and medians of the respective dataset.

To evaluate RPE mitochondrial structure and subcellular distribution as a function of mouse genetic background, we first performed live imaging of Mitotracker deep red in RPE flatmounts generated from wildtype pigmented C57BL/6J, pigmented 129S1/SvlmJ, and albino BALB/c mice. Our data show striking differences in mitochondrial morphology and distribution between pigmented and albino mice RPE. Whereas mitochondria in pigmented C57BL/6J and 129S1/SvlmJ RPE form interconnected networks along the cell periphery, mitochondria in albino BALB/c RPE mice were largely fragmented and appear as disconnected discrete structures (Figures 5A,B). To establish that this was not an artifact of live imaging, we immunostained RPE flatmounts for TOM20 and observed a similar pattern of smaller mitochondrial volumes in albino RPE compared to that of pigmented RPE (Figures 5C,D, Supplementary Movies S6-8). Comparison of live imaging and fixed imaging datasets revealed that although fixation resulted in decreased mean mitochondrial volumes in all three mouse strains (similar to that observed in primary RPE cultures in Figure 1), differences between mouse strains are conserved and correlate with live imaging data (Figure 5D). Analyses of mitochondrial distribution as a function of z-position within the RPE revealed another important difference between mouse strains: unlike mitochondria in pigmented RPE that are distributed throughout the volume of the cell, mitochondria in albino RPE are predominantly concentrated near the basal surface of the RPE (Figure 5E).

**FIGURE 6:**
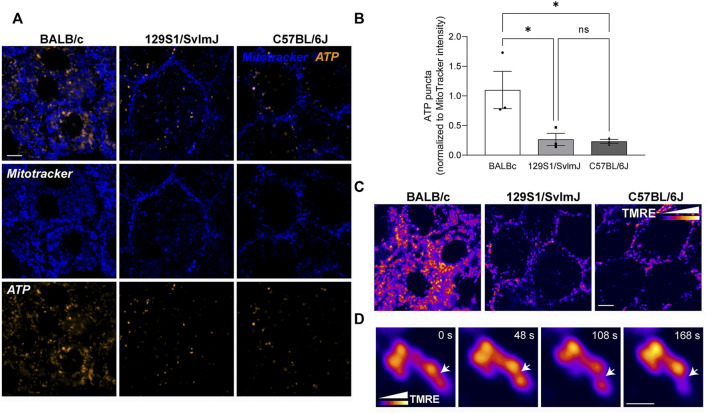
Genetic background of wildtype mice determines mitochondrial integrity and function in the RPE. **(A)** Stills from live imaging of mitochondria (Mitotracker, blue) and BioTracker-ATP (gold) in albino BALB/c, pigmented 129S1/SvlmJ, and pigmented C57BL/6J mouse RPE flatmounts. **(B)** Quantitation of ATP puncta normalized to Mitotracker intensity. Mean ± SEM. *n* = 3 mice per strain. *p = 0.0497 between BALB/c and 129S1/SvlmJ; *p* = 0.0427 between BALB/c and C57BL/6J by one-way ANOVA. **(C)** Mitochondrial membrane potential (MMP) measured by TMRE labeling in mouse RPE flatmounts. Color scale depicts relative intensity of TMRE. Scale bar = 3 µM. **(D)** High magnification images showing local heterogeneity of MMP within a single mitochondrion. Note that areas of low TMRE intensity predict putative fission sites (arrow). Scale bar = 1 µM. Also see Supplementary Movies S9.

We then asked if the significant differences in RPE mitochondrial morphology and distribution translate to functional differences in albino and pigmented mice. We focused on mitochondrial ATP and ΔΨm as functional readouts of mitochondrial health and bioenergetics. Live imaging showed higher BioTracker ATP signal in albino mouse RPE flatmounts compared to pigmented RPE (Figures 6A,B), suggesting that albino RPE have higher ATP content compared to pigmented RPE. Of note, although the BioTracker ATP signal was in close proximity to the mitochondria, it did not colocalize strongly with Mitotracker fluorescence (Figure 6A) unlike that reported in the oral squamous cell carcinoma cells in which the probe was first tested (Wang et al., 2016). This potentially reflects cell-type-specific differences in cellular bioenergetics. Because interconnected mitochondria are more efficient in oxidative phosphorylation compared to fragmented mitochondria (Chen et al., 2005; Gomes et al., 2011; Rambold et al., 2011), it is also plausible that the increased BioTracker ATP signals we observed in the albino RPE reflects ATP generated *via* glycolysis in the cytosol (Ozawa et al., 2015). Further studies are required to establish how functional mitochondria are in the albino RPE, and whether there is a shift from oxidative phosphorylation towards glycolysis in albino RPE due to increased oxidative stress and mitochondrial fragmentation.

Live imaging of TMRE staining in the RPE of albino and pigmented mice showed heterogeneity of ΔΨm within individual mitochondrial similar to our data in primary RPE monolayers (Figure 6C). High-speed live imaging allowed us to capture focal loss of ΔΨm preceding mitochondrial fission in 129S1/SvlmJ mouse RPE flatmounts (Figure 6D, Supplementary Movies S9), consistent with our observation in primary RPE cultures (Figure 3C).

## Discussion

The unique features of the RPE–a postmitotic, polarized tissue that deals with the immense metabolic burden of daily photoreceptor outer segment clearance in a highly oxidative environment–places unique stresses on its mitochondria. Recent studies show that mitochondrial function influences RPE cell fate (Hazim et al., 2022), and that AMD-associated stressors such as smoking and complement activation promote mitochondrial fragmentation and dysfunction (Woodell et al., 2013; Tan et al., 2016; La Cunza et al., 2021). Given that RPE mitochondrial function directly impacts photoreceptor health and vision (Handa et al., 2019; Ferrington et al., 2021; Hurley, 2021), insight into mechanisms and machinery that maintain mitochondrial homeostasis in the RPE *in vivo* and how they are disrupted in disease is essential for developing sight-saving therapies.

Mitochondrial form is closely related to its function (Liesa and Shirihai, 2013; Giacomello et al., 2020). Early studies using two-dimensional electron microscopy showed mitochondria as discrete sausage-shaped structures in the cell. Over the last decade or so, advances in high-resolution and super-resolution microscopy have allowed imaging of organelles below the diffraction limit of light (250 nm laterally and 500 nm axially). These studies show that mitochondria form extensive networks that span the volume of the cell and dynamically interact with the ER, lysosomes, and other organelles (Murley and Nunnari, 2016; Iannetti et al., 2019; Lackner, 2019; Giacomello et al., 2020; Aoyama-Ishiwatari and Hirabayashi, 2021; Cisneros et al., 2022). Using these advanced imaging tools to investigate mitochondrial dynamics in the RPE is complicated by technical challenges in dealing with RPE polarity, pigmentation, and accumulation of lipofuscin. For instance, super-resolution techniques such as Structured Illumination Microscopy (SIM) is compatible with imaging pigmented cells, while Stimulated Emission Depletion (STED) or Stochastic Optical Reconstruction Microscopy (STORM) cannot be used to image pigmented RPE because melanin interferes with fluorophore quenching and photo-switching of the dyes. Imaging mitochondrial dynamics in living cells requires balancing image resolution with acquisition speed to capture fission, fusion, and contact sites. In this regard, capturing and processing multiple images per plane required for SIM, slows down the acquisition speed especially for multicolor imaging, which prevents accurate temporal analysis of mitochondrial dynamics.

Here, we used a spinning disc confocal microscope with an EMCCD camera engineered for very fast acquisition (26 fps) coupled with the Live-SR super-resolution module, which increases image resolution to ∼120–140 nm laterally without any loss of acquisition speed. Imaging mitochondrial dynamics in polarized primary RPE cultures and mouse RPE flatmounts using this system showed that mitochondrial networks in the RPE reflect its differentiation status and are destroyed by fixation. Therefore, experiments on mitochondrial function in fixed non-polarized or poorly differentiated RPE cultures should be evaluated carefully. The increased resolution and acquisition speeds enabled by our system showed that RPE mitochondria show localized heterogeneities in membrane potential and ATP production that could reflect focal changes in metabolism and oxidative stress; that contacts between the mitochondria and ER modulate calcium flux; and that lysosomes remodel the mitochondrial network by mediating fission.

In comparing mitochondrial dynamics in RPE flatmounts from widely used mouse strains, we unexpectedly observed that in contrast to the highly interconnected mitochondria in the RPE of pigmented mouse strains, mitochondria in albino mouse RPE are highly fragmented and localized to the basal surface of the RPE. The difference in mitochondrial structure may contribute to the increased susceptibility of albino rodents to AMD-like pathology upon light damage, including changes in photoreceptor function, photoreceptor death, and accumulation of subretinal deposits (Montalbán-Soler et al., 2012; Polosa et al., 2016). Moreover, albino *Abca4* ^*−/−*^ mouse model of inherited macular degeneration exhibits progressive photoreceptor loss beginning at 8 months of age, whereas no photoreceptor death was observed up to 18 months of age in pigmented *Abca4* ^*−/−*^ mice (Radu et al., 2008; Charbel Issa et al., 2013; Wu et al., 2014). The link between albino RPE and mitochondrial dysfunction is likely mediated by several factors: First, melanosomes in pigmented RPE act as anti-oxidants to sequester reactive oxygen species, thereby protecting the RPE against mitochondrial DNA damage and disruption of mitochondrial respiratory chain activity (Seagle et al., 2005; Van Houten et al., 2006; Rózanowski et al., 2008; Voets et al., 2012). Secondly, light exposure promotes photodegradation of lipofuscin bisretinoids in the RPE resulting in increased RPE oxidative stress, that is, especially pronounced in albino mice (Ueda et al., 2016). Alteration in cellular redox state could also directly modulate mitochondrial integrity by promoting redox-sensitive post-translational modifications in proteins involved in mitochondrial fission and fusion (Willems et al., 2015).

Interconnected mitochondria are known to be more efficient at OXPHOS and ATP generation compared to fragmented mitochondria (Chen et al., 2005; Gomes et al., 2011; Rambold et al., 2011). However, we observed increased ATP fluorescence in albino mice RPE compared to pigmented RPE. Because the ATP signal did not colocalize with Mitotracker, we hypothesize that OXPHOS defects in fragmented mitochondria could lead to increased glycolysis in albino RPE resulting in ATP generation in the cytosol. Moreover, although fragmented mitochondria are defective in OXPHOS, this does not always translate to loss of ATP production, because these mitochondria can consume alternate fuel sources such as aspartate to generate ATP (Yao et al., 2019). Bioenergetic profiling of albino and pigmented mouse RPE using SeaHorse Flux Analyzer or commercially available kits to measure temporal changes in oxygen consumption rates (Hazim et al., 2022) will provide valuable insight into bioenergetic differences in these mice. While mechanisms responsible for the observed increased ATP production in albino RPE need further investigation, one negative consequence would be the accelerated generation of reactive oxygen species (ROS), which would exacerbate oxidative stress in albino RPE (Rattner et al., 2008; Hunter et al., 2012; Mao et al., 2014; Brown et al., 2019). This is supported by an intriguing study showing that temporal RPE in pigmented C57BL/6J mice have fewer melanosomes and are more susceptible to oxidative stress (Dieguez et al., 2019). Since mitochondrial cristae harbor OXPHOS machinery, high-resolution live imaging of mitochondrial cristae organization (Segawa et al., 2020) and ROS generation (Chandrasekharan et al., 2019) in pigmented and albino mice is necessary for better insight into the mechanisms involved.

Based on electron microscopy studies of mouse, non-human primate, and human RPE, it has been widely thought that mitochondria are predominantly located basally where there is a large energy demand (Gouras et al., 2010; Williams et al., 2013; Gouras et al., 2016; Pollreisz et al., 2020). However, a recent serial block-face scanning EM analysis of a single human donor shows apical mitochondria (Pollreisz et al., 2020). As we show in this study, fixation can induce artifacts that can drastically alter mitochondrial morphology and distribution in the RPE. Our high-resolution live imaging of pigmented mouse RPE flatmounts now show that RPE mitochondria are spread throughout the volume of the cell with a band of highly integrated mitochondria near the circumferential actin ring below the junctional complexes, which has also been observed by others (McWilliams et al., 2019). These interconnected mitochondria could serve multiple functions for RPE homeostasis such as maintaining cell-cell junctions that depend on ATP (Hong et al., 2010); promoting ATP release from gap junctions to support development of the neural retina (Pearson et al., 2005); and providing ATP for the phagocytosis and degradation of photoreceptor outer segments, an energetically intensive function. In albino mice RPE, lack of this band of interconnected mitochondria could explain the observed gap junction and adherens junction abnormalities (Iwai-Takekoshi et al., 2016) and delayed retinal neurogenesis (Bhansali et al., 2014).

Currently, imaging mitochondrial dynamics in human RPE is limited by access to donor tissues soon after death and the presence of lipofuscin that can interfere with imaging. Autofluorescence quenching tools such as TrueBlack can be used for both fixed and live imaging human donor RPE (Kaur et al., 2018; La Cunza et al., 2021). Development of new imaging technologies such as three-photon microscopy that can image pigmented RPE or increased resolution of adaptive optics optical coherence tomography (AO-OCT) would allow monitoring mitochondrial dynamics in the living eye (Liu et al., 2016; Shirazi et al., 2020; Bower et al., 2021).

This study demonstrates the power of high-speed, high-resolution live imaging for evaluating the integrity, health, and function of mitochondria in fully differentiated RPE monolayers in culture and in mouse RPE flatmounts. We show that live imaging is essential for investigating spatiotemporal changes in mitochondrial fusion and fission, as well as changes in mitochondrial membrane potential and ATP generation that are not detectable in fixed tissues. A key finding from this study is the striking difference in mitochondrial integrity and localization between albino and pigmented mice RPE. Albino mice have been used to generate transgenic models of retinal disease because of the ease of imaging RPE without pigment and because their increased susceptibility to oxidative stress shortens the time to retinal degeneration compared to transgenic mice on a pigmented background (Ueda et al., 2016). Our data now show that it is important to compare mitochondrial integrity and dynamics in wildtype mice of different genetic strains to better understand the impact of the gene of interest in transgenic models. We anticipate that these studies will provide a framework to guide experimental design and selection of mouse models for evaluating RPE mitochondrial function in health and disease.

## Data Availability

The original contributions presented in the study are included in the article/Supplementary Material, further inquiries can be directed to the corresponding author.
