# Supplementary material for: Analysis of mitochondrial dynamics and function in the retinal pigment epithelium by high-speed high-resolution live imaging
Source: Front Cell Dev Biol. 2022 Oct 28;10:1044672. doi: 10.3389/fcell.2022.1044672 (PMC9651161; doi:10.3389/fcell.2022.1044672)
Supplement: Supplementary file 4 [file DataSheet1.PDF]

Supplementary Material

Movie Caption

Movie 1. Live imaging of mitochondrial dynamics (Mitotracker Deep Red, red) in primary polarized RPE monolayer using ~2 s timelapse interval for a total imaging time of 2 min.

Movie 2. Live imaging of mitochondrial dynamics (Mitotracker Deep Red, red) in primary polarized RPE monolayer using ~15 s timelapse interval for a total imaging time of 2 min.

Movie 3. Live imaging of changes in mitochondrial membrane potential (measured by TMRE, left) in a mitochondrial scission event (Mitotracker Deep Red, right) in a primary RPE monolayer. Warmer colors indicate higher fluorescence intensity of TMRE.

Movie 4. Live imaging of a dynamic mitochondrial calcium (mito-GCaMP5G, cyan) transfer event between mitochondrial strands (MitoTracker Deep Red, blue) mediated by the ER (ER-Tracker, red).

Movie 5. Live imaging of interactions between mitochondria (Mitotracker Deep Red, red) and lysosomes (LysoTracker, green) during a mitochondrial fission event.

Movie 6. Immunostaining of TOM20 (red) in an RPE flatmount from an albino BALB/c mouse. Animation plays through the RPE height starting from the apical surface, progressing to the basal side of the RPE, and then reversing back to the topmost apical plane. Actin is stained with phalloidin (white), and nuclei are labeled with DAPI (blue).

Movie 7. Immunostaining of TOM20 (red) in an RPE flatmount from a pigmented 129S1/SvlmJ mouse. Animation plays through the RPE height starting from from the apical surface, progressing to the basal side of the RPE, and then reversing back to the topmost apical plane. Actin is stained with phalloidin (white), and nuclei are labeled with DAPI (blue).

Movie 8. Immunostaining of TOM20 (red) in an RPE flatmount from a pigmented C57BL/6J mouse. Animation plays through the RPE height starting from from the apical surface, progressing to the basal side of the RPE, and then reversing back to the topmost apical plane. Actin is stained with phalloidin (white), and nuclei are labeled with DAPI (blue).

Movie 9. Live imaging of mitochondrial membrane potential (TMRE) in a live RPE flatmount from 129S1/SvlmJ mouse. Warmer colors represent higher TMRE intensities.

Supplementary Table S1. List of reagents and sources.

Antibody	Host	IF	Source	Identifier
TOM20	Rabbit	1:200	Santa Cruz Biotechnology Inc., Dallas, Texas	sc-11415
CoraLite-conjugated TOM20	Rabbit	1:200	ProteinTech, Rosemont, IL	CL488-11802
Alexa Fluor 568 anti-Rabbit secondary antibody	Donkey	1:500	ThermoFisher Scientific, Waltham, MA	A10042

Dyes	Concentration	Source	Catalog number
DAPI	14.3 mM stock solution, used at 1:200	Sigma-Aldrich, St. Louis, MO	D9542
Acti-stain 488 phalloidin	1:200, incubated with secondary antibodies	Cytoskeleton Inc., Denver, CO	PHDG1
Lysotracker Red	200 nM, 15 min	ThermoFisher, Waltham, MA	L7528
Mitotracker Deep Red	200 nM, 15 min	ThermoFisher, Waltham, MA	M22426
ER-tracker Red	1 µM, 30 min	ThermoFisher, Waltham, MA	E34250
TMRE	500 nM, 10 min	Biotium, Fremont, CA	70005

Plasmid	Ref	Source	Identifier
pCAG mito-GCaMP5G	(1)	Addgene, Watertown, MA	105009

Biochemicals	Source	Identifier
Bovine Serum Albumin	Rockland Immunochemicals, Pottstown, PA	BSA-50
Calcium Chloride Dihydrate	Sigma-Aldrich, St. Louis, MO	C7902

Glucose	Sigma Aldrich, St. Louis, MO	G7528
HBSS	Corning, Corning, NY	21-023-CV
HEPES	ThermoFisher, Waltham, MA	15630080
Magnesium Chloride Hexahydrate	Sigma-Aldrich, St. Louis, MO	M2393
Paraformaldehyde (8%)	Electron Microscopy Sciences, Hatfield, PA	157-8
Phosphate Buffer Saline	ThermoFisher Scientific, Waltham, MA	BP665-1
Saponin	Sigma-Aldrich, St. Louis, MO	84510
Triton-X	Sigma-Aldrich, St. Louis, MO	X100-500ML
VectaShield	Vector Laboratories, Burlingame, CA	H1000

**Components for porcine RPE cell culture**		
Ciprofloxacin	Sigma-Aldrich, St. Louis, MO	17850-5G-F
DMEM	Corning, Corning, NY	10-013-CV
Fetal Bovine Serum (heat-inactivated)	American Type Culture Collection, Manassas, VA	30-2020
Non-essential amino acids (NEAA)	Corning, Corning, NY	25-025-CI
Penicillin-Streptomycin	Corning, Corning, NY	30-002-CI
0.25% Trypsin	Corning, Corning, NY	25-053-CI
2.5% Trypsin	Lonza, Walkersville, MD	17-160E
Opti-MEM	Gibco, ThermoFisher, Waltham, MA	319t85-070

References

1. Kwon SK, Sando R, Lewis TL, Hirabayashi Y, Maximov A, Polleux F. LKB1 Regulates Mitochondria-Dependent Presynaptic Calcium Clearance and Neurotransmitter Release Properties at Excitatory Synapses along Cortical Axons. PLoS Biol. 2016;14(7):e1002516.
